# Supplementary material for: The interdependence of excitation and inhibition for the control of dynamic breathing rhythms
Source: Nat Commun. 2018 Feb 26;9:843. doi: 10.1038/s41467-018-03223-x (PMC5827754; doi:10.1038/s41467-018-03223-x)
Supplement: Supplementary file 1 — Supplementary Information [file 41467_2018_3223_MOESM1_ESM.pdf]

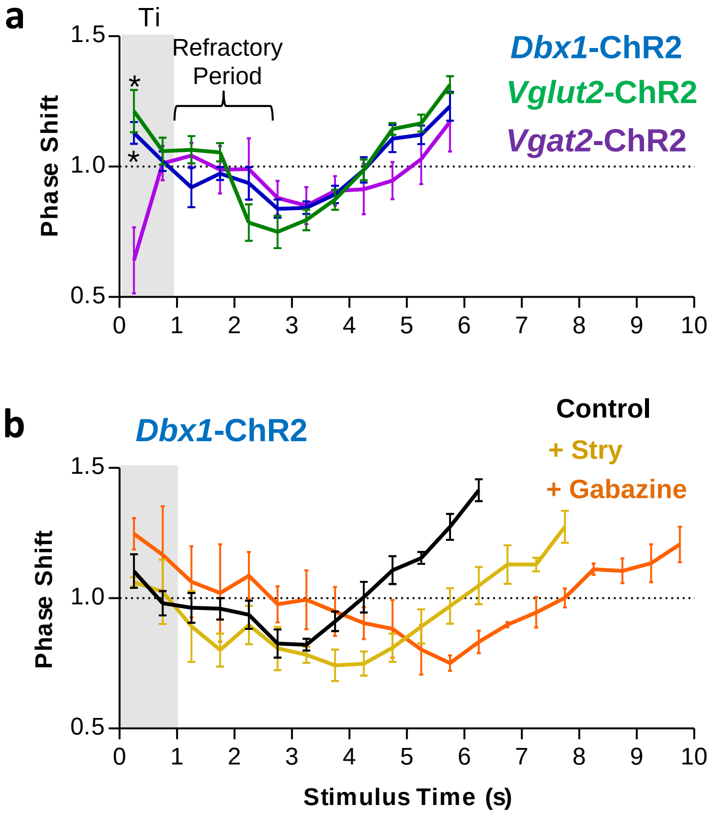

Supplementary Fig. 1 Comparisons of light-induced respiratory phase-shift in vitro. **a** Average phase-shift (stimulus cycle duration/average endogenous cycle duration) elicited relative to the time delay between the light stimulus and the preceding inspiratory burst in brainstem slices from *Dbx1*-ChR2 (n=7), *Vglut2*-ChR2 (n=7), and *Vgat*-ChR2 (n=7) mice (means±s.e.m., two-way ANOVA and Bonferroni's multiple comparisons test, *p<0.0001 compared to *Vgat*-ChR2). Note the differential effects of stimulating excitatory (Dbx1/Vglut2) vs inhibitory (Vgat) populations during the inspiratory burst (Ti). Also note the similar effects during the refractory period. **b** Average data from Fig. 2c showing the phase-shift evoked by *Dbx1*-ChR2 stimulation in n=4 brainstem slices under control conditions (black) and in strychnine (yellow) and strychnine+gabazine (orange) (means±s.e.m.). Note the increased duration of the refractory period and expiratory time following blockade of synaptic inhibition.

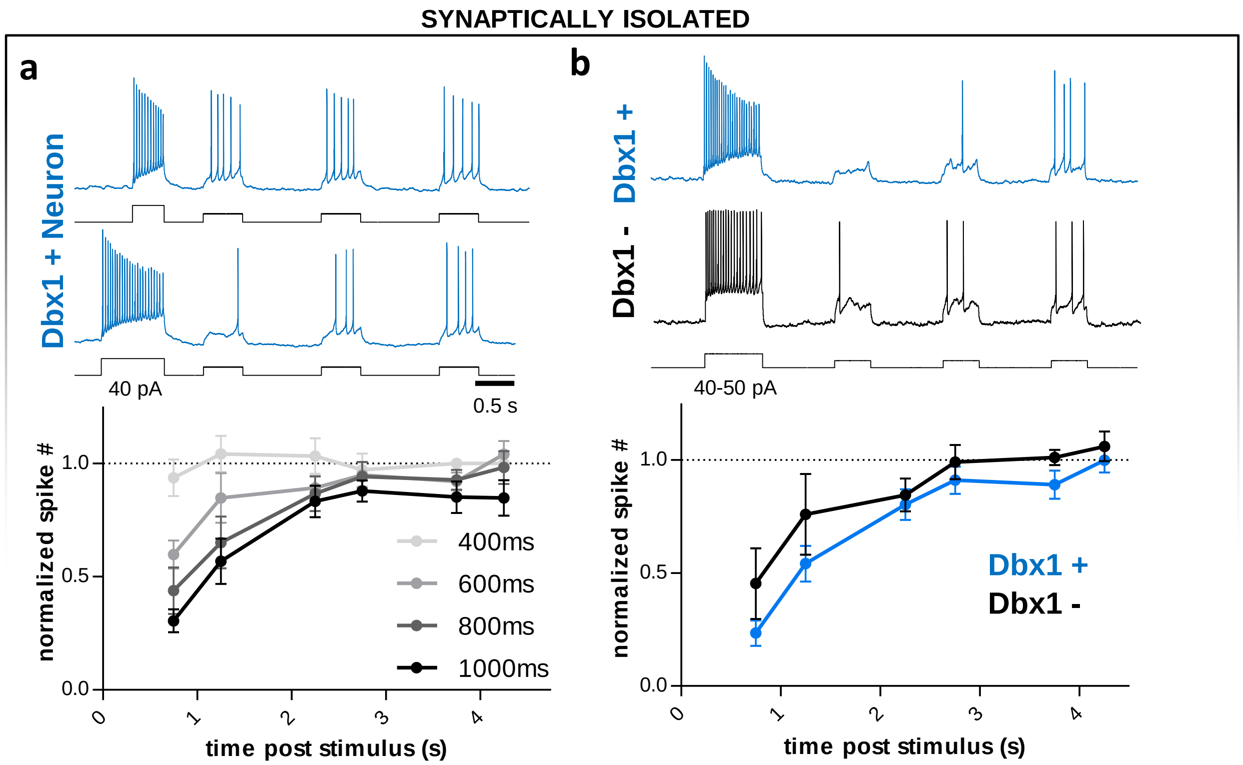

Supplementary Fig. 2 An intrinsic refractory period for preBötC neurons. **a** Representative evoked activity of a synaptically isolated Dbx1+ neuron following a short (400ms) and long (800ms) current step (40pA), and average normalized number of spikes evoked relative to elapsed time following varied stimulus durations in n=7 Dbx1+ neurons. **b** Representative evoked activity of synaptically isolated Dbx1+ and Dbx1- neurons following a 40-50pA simulated drive potential, and average normalized spike number comparing the intrinsic refractory period for Dbx1+ (n=8) and Dbx1- (n=6) neurons (means±s.e.m., two-way repeated measures ANOVA and Bonferroni's post-hoc test; p>0.05). Scale bars=0.5s

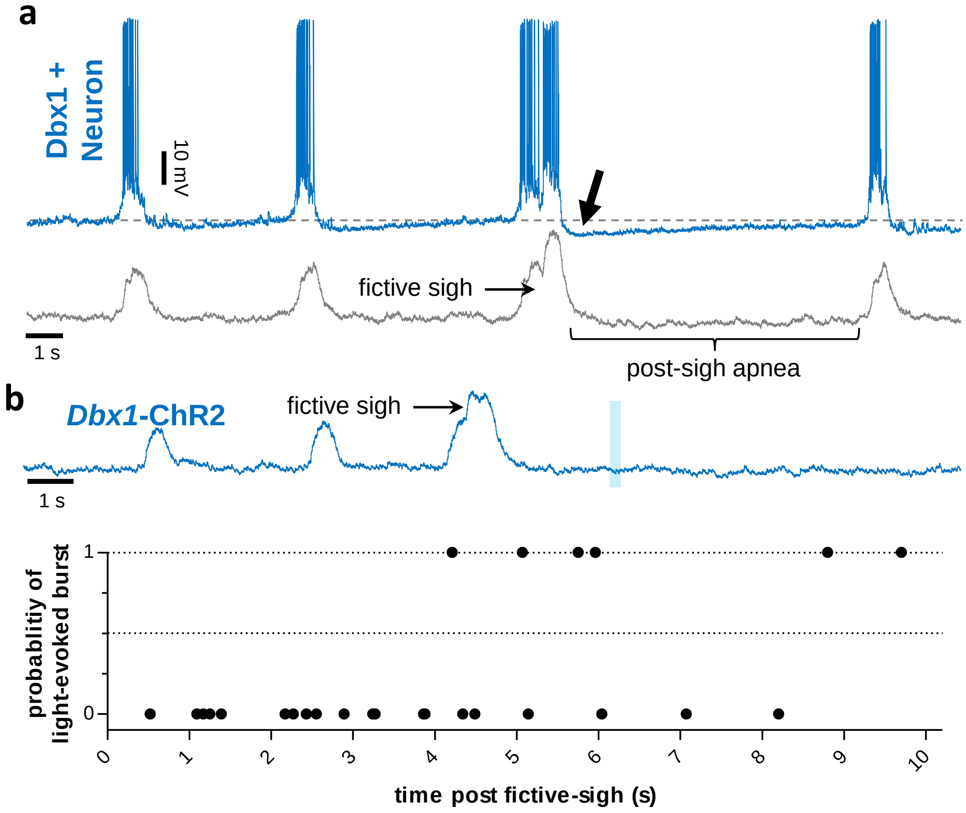

Supplementary Fig. 3 The refractory period is exaggerated following sigh bursts in the preBötC, easily distinguished by their biphasic shape, with a large burst superimposed on an initially normal "eupneic" burst. **a** Representative whole-cell recording of a Dbx1+ neuron and concurrent preBötC population activity during a fictive-sigh. Arrow indicates larger after-hyperpolarization of Dbx1+ neuron membrane potential and associated elongation of Te (post-sigh apnea) following the fictive sigh. **b** Representative recording from a *Dbx1*-ChR2 slice showing failure to evoke a preBötC burst with light stimulation >2s following a fictive sigh (top), and probability of evoking a preBötC burst as a function of time following fictive-sighs (bottom). Points represent individual light stimulations.

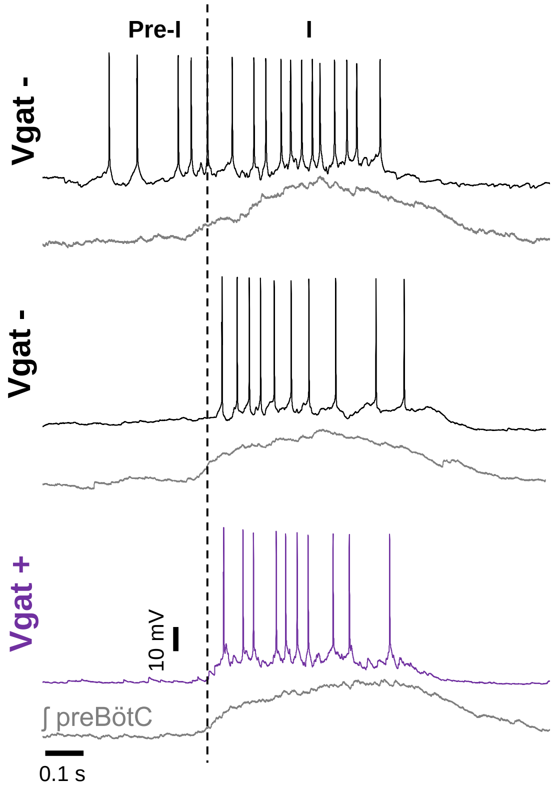

Supplementary Fig. 4 Inhibitory preBötC neurons do not exhibit pre-inspiratory activity. Whole-cell recordings and concurrent integrated preBötC population activity from representative inspiratory neurons identified as Vgat- or Vgat+. In a subset of Vgat- neurons, spiking began before the onset of inspiration and continued for the duration of the inspiratory burst. In all 14/14 recorded inspiratory Vgat+ neurons, spiking activity was isolated during preBötC bursts, indicating a lack of pre-I activity.

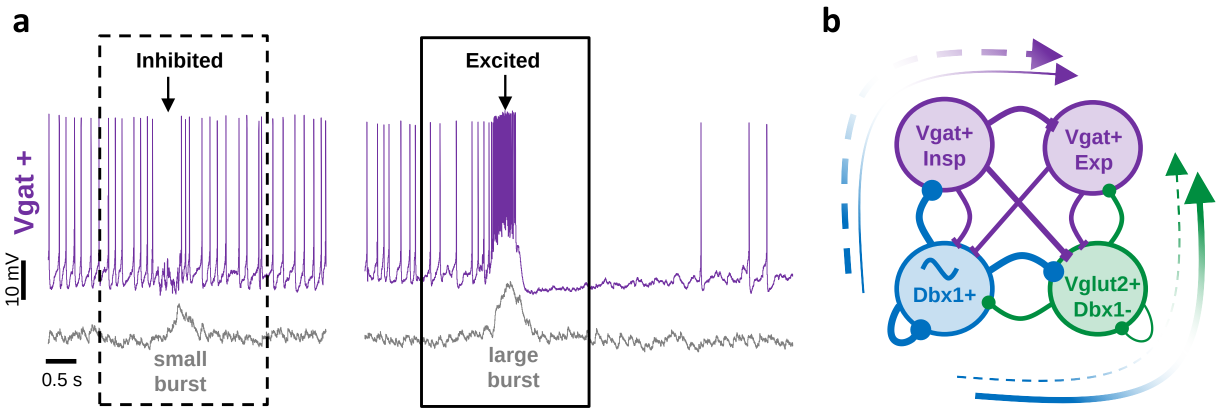

Supplementary Fig. 5 Inhibitory neurons active during expiration receive concurrent excitation and inhibition. **a** Representative Vgat+ neuron that is inhibited during small preBötC population bursts (dashed line), but excited during large amplitude bursts (solid line). **b** Hypothesized excitatory and inhibitory connections to expiratory Vgat+ neurons demonstrating pathways leading to a switch from dominant inhibitory inputs during small bursts (dashed lines) to dominant excitatory inputs during large bursts (solid lines).

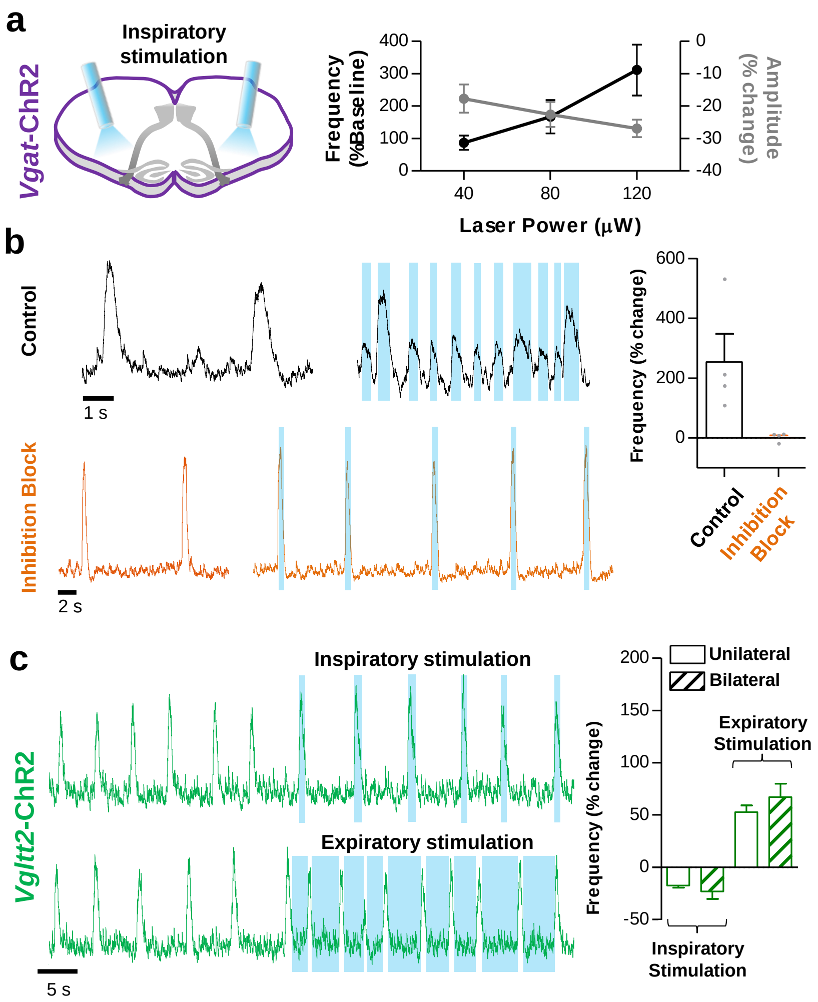

Supplementary Fig. 6 Phase dependent stimulations of excitatory and inhibitory preBötC neurons. **a** Laser power-dependent changes in preBötC burst frequency and amplitude during bilateral laser stimulation in n=4 *Vgat*-ChR2 brainstem slices (means+s.e.m.). Changes in frequency and amplitude were negatively correlated ($p<0.05$, linear regression analysis). **b** Example recording from a *Vgat*-ChR2 brainstem slice during inspiratory stimulation under control conditions (black) and following blockade of synaptic inhibition (orange) with strychnine (1μM) and gabazine (1μM). Average data from n=4 slices are shown on the right. Note that the effects of *Vgat*-ChR2 stimulation are lost following blockade of inhibition. **c** Representative examples of inspiratory and expiratory specific contralateral light stimulation of the preBötC in a *Vglut2*-ChR2 slice. Average changes in burst frequency during contralateral (open bars) and bilateral (patterned bars) stimulation (right) (means+s.e.m.).

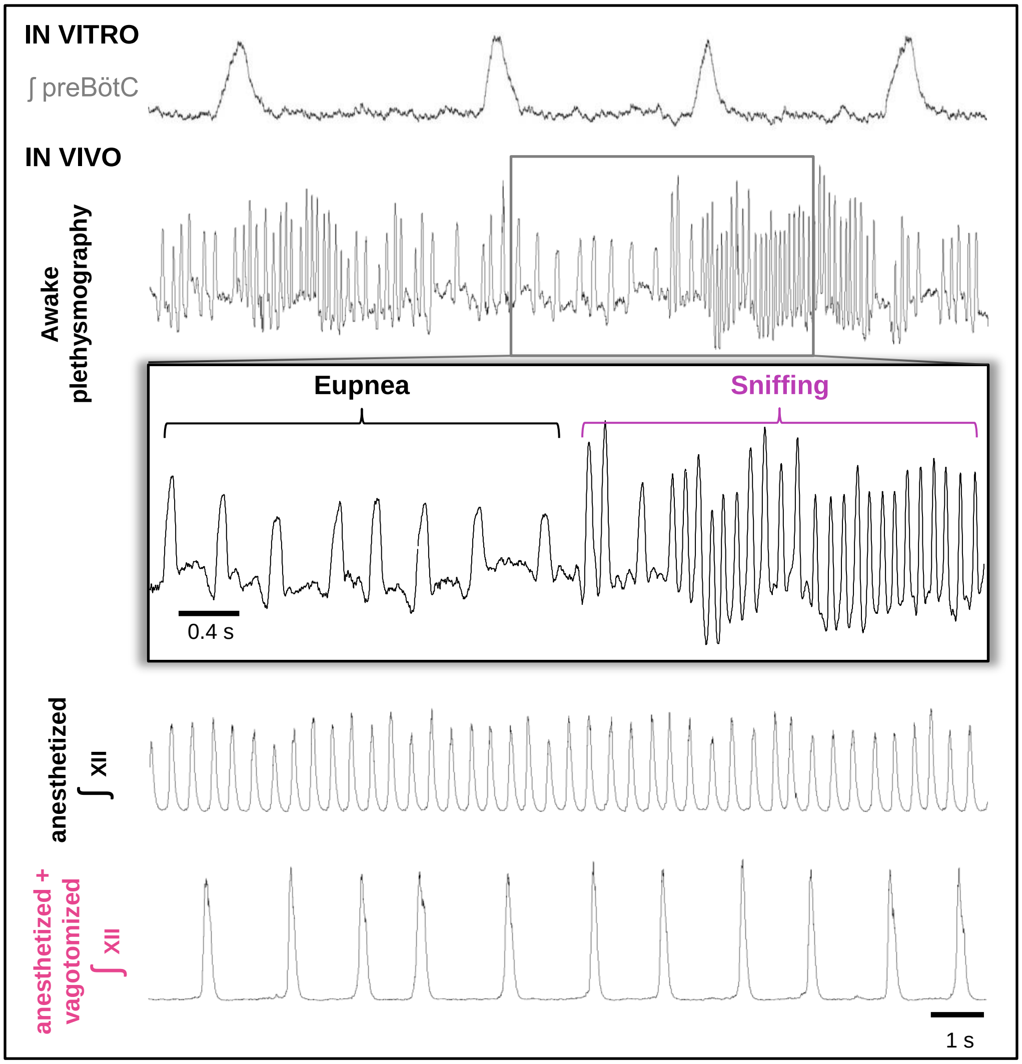

Supplementary Fig. 7 Breathing patterns in intact and reduced mouse preparations. Comparison of representative inspiratory activity recorded in vitro from the preBötC transverse slice preparation, and in vivo awake, anesthetized, and anesthetized and vagotomized mice. Expanded trace of inspiratory activity in an awake mouse shows the transition from eupnea to sniffing.

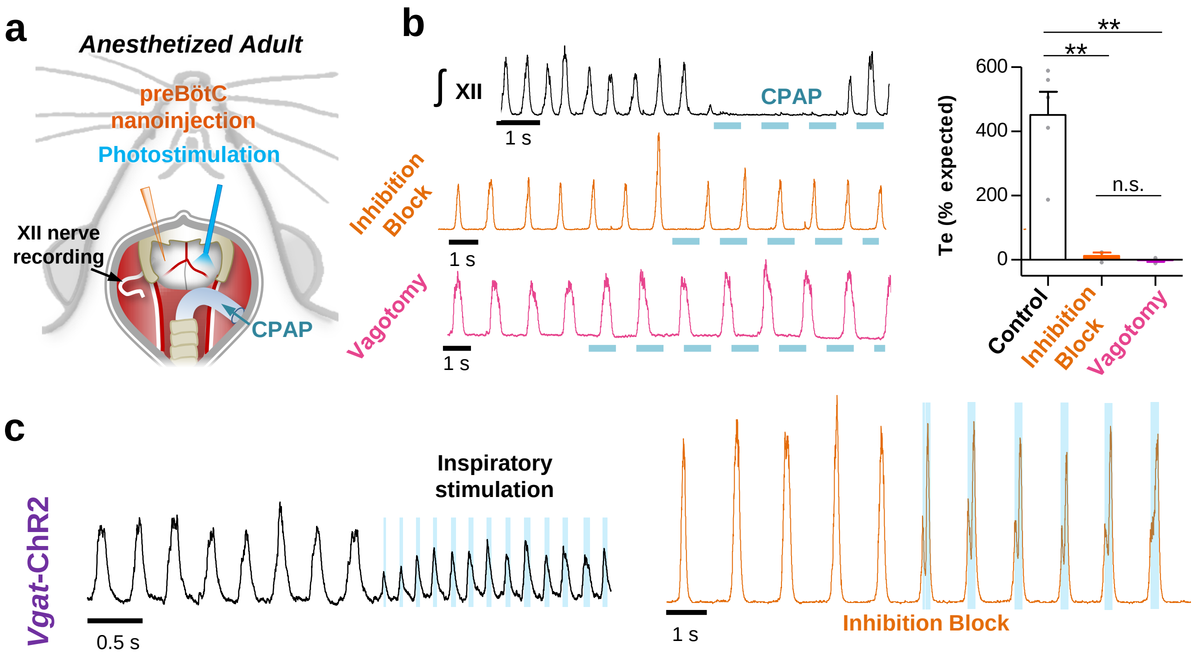

Supplementary Fig. 8 Effects of *Vgat*-ChR2 stimulation and vagal feedback are mediated by inhibition local to the preBötC. **a** Schematic depicting the approach to examine the vagally-mediated Breuer-Hering reflex by applying continuous positive airway pressure (CPAP) to induce lung stretch in anesthetized adult mice. **b** Representative XII nerve activity during CPAP under control conditions (black), following bilateral preBotC injection of strychnine+gabazine (orange), or following vagotomy (pink). Average data are shown on the right (control, n=5; inhibition block, n=3; vagotomy, n=3) (means+s.e.m., one-way ANOVA and Bonferonni's multiple comparisons test, $**p<0.01$, n.s., not significant). **c** Example XII nerve activity during inspiratory light stimulation in an anesthetized *Vgat*-ChR2 mouse before (black) and after bilateral preBotC injection of strychnine+gabazine (orange).

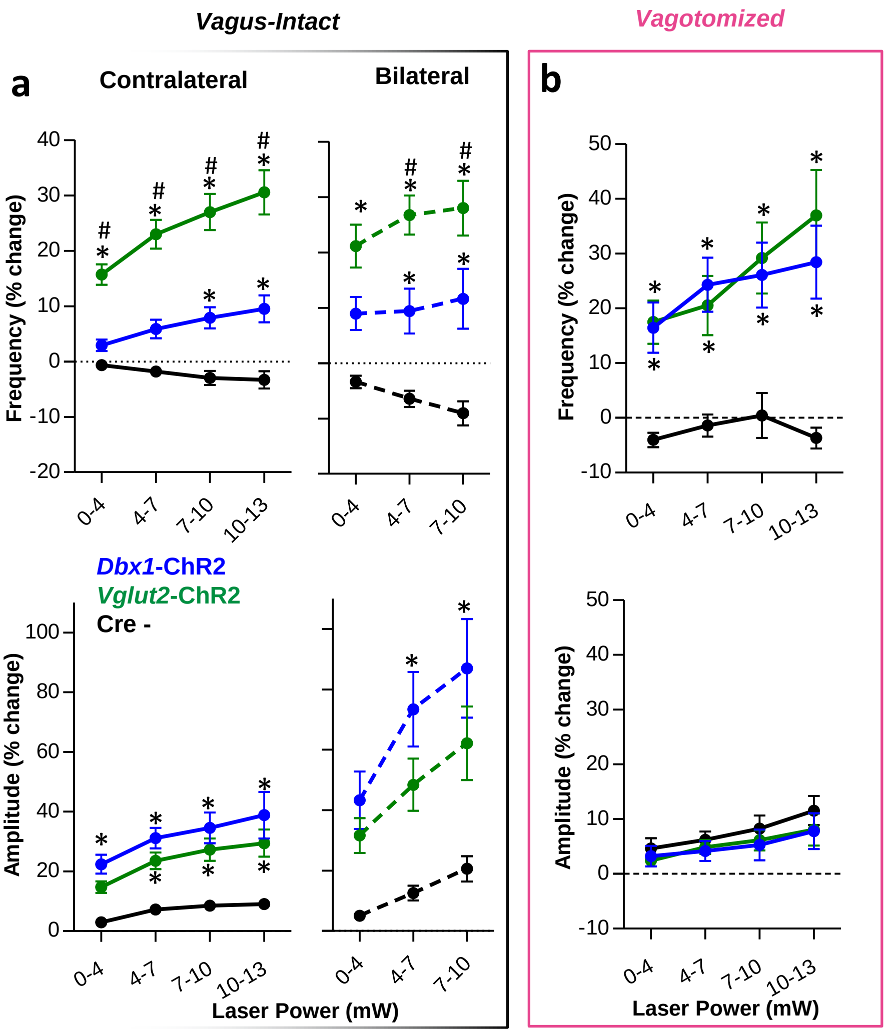

Supplementary Fig. 9 Laser power-dependent effects on breathing during activation of overlapping excitatory preBötC populations in vivo. **a** Average change XII nerve burst frequency and amplitude during contralateral (Dbx1, n=10; Vglut2, n=9; Cre-, n=10) or bilateral (Dbx1, n=9; Vglut2, n=8; Cre-, n=7) continuous (10s) light stimulation across increasing laser powers in vagus-intact mice. Note that the differential, frequency-specific, effects of *Dbx1*-ChR2 vs. *Vglut2*-ChR2 stimulation are maintained during increasing laser power. **b** Average change in XII burst frequency and amplitude during continuous light stimulation at different laser powers in vagotomized mice (Dbx1, n=9; Vglut2, n=8; Cre-, n=9). means±s.e.m., two-way repeated measures ANOVA and Bonferonni's post hoc test, *p<0.05 compared to Cre- control, #p<0.05 compared to *Dbx1*-ChR2.
